# The effect of parental achievement pressure and self-regulated learning on school adjustment, mediated by self-esteem

**DOI:** 10.3389/fpsyg.2026.1751186

**Published:** 2026-03-24

**Authors:** Sun-Geun Baek, Kai Zhang

**Affiliations:** Department of Education, Seoul National University, Seoul, Republic of Korea

**Keywords:** parental achievement pressure, school adjustment, self-esteem, self-regulated learning, structural equation modeling

## Abstract

**Introduction:**

This study aimed to conduct an analysis using structural equation modeling (SEM) to identify the structural relationship between parental achievement pressure and students’ self-regulated learning and their influence on school adjustment through self-esteem.

**Method:**

This study used data from the 14th Korean Children’s Panel (2021) to examine the effects of parental achievement pressure and self-regulated learning on school adjustment, and to verify the mediating role of self-esteem. Among 2,150 middle school students aged 13, missing values were identified, and a total of 1,328 responses were included in the final analysis. Confirmatory factor analysis (CFA) was conducted to assess the validity of the measurement model and the overall model fit. Structural equation modeling (SEM) was employed to examine the structural relationships among the variables, as well as to assess the direct, indirect, and total effects along the paths.

**Results and discussion:**

Parental achievement pressure was found to have a significant negative effect on school adjustment. This indicates that higher parental expectations regarding academic performance can adversely affect a child’s adjustment to school. Moreover, excessive parental expectations regarding academic achievement can impose psychological and academic stress on children, leading to lower self-esteem. Self-regulated learning was also found to significantly influence self-esteem, and the mediating role of self-esteem in the effects of parental achievement pressure and self-regulated learning on school adjustment was statistically significant. Specifically, when parents place greater emphasis on academic outcomes, middle school students may experience increased academic stress and emotional burden, resulting in decreased self-esteem, which in turn negatively impacts school adjustment. Conversely, students with strong self-regulated learning skills tend to have higher self-esteem, as they experience a sense of accomplishment by setting their own learning goals, overcoming challenging problems, and managing stress effectively. Therefore, it is necessary to implement educational programs for parents, such as promoting positive parenting attitudes, and to develop support and mentoring programs aimed at enhancing children’s self-esteem.

## 1 Introduction

In recent years, adolescents’ school adjustment has increasingly attracted scholarly attention, underscoring its significance within both pedagogical theory and psychological research. Adolescence is widely recognized as a critical developmental stage characterized by rapid physical, emotional, and social changes, during which academic achievement and successful adjustment to school life are pivotal for individual growth and social integration. Within the educational context, active engagement in academic activities, the establishment of positive peer relationships, and effective communication with teachers have been identified as essential components that substantially contribute to adolescents’ holistic development.

School adjustment refers to the active process through which students meet the demands of the school environment—complying with rules, acquiring knowledge and skills, and maintaining positive relationships with peers and teachers (Lee and Kim, 2008). Prior research has consistently demonstrated that school adjustment significantly influences adolescents’ academic achievement, psychological stability, and even their long-term developmental trajectories (Kim and Hong, 2019; Jung and Ahn, 2023). Furthermore, as scholarly attention has increasingly turned toward the ecological factors shaping adolescents’ lives, a growing body of research has sought to examine the influence of various environmental contexts—including the home, school, and community—on youth development (Park and Eom, 2012). While such pressure may initially catalyze academic motivation, it frequently generates debilitating stress and anxiety when it becomes excessive (Woo, 2021).

Previous studies have actively examined variables influencing adolescents’ school adjustment. Among parental factors, parenting style, autonomy support, achievement pressure, empathy, and attachment have been highlighted (Choi and Yeon, 2022; Hong and Rho, 2022; Lee and Cho, 2021; Park, 2021; Jeong and Ahn, 2023a). Alongside these, individual factors such as self-regulated learning, self-esteem, and career maturity have also shown strong associations (Jeong and Ahn, 2023b; Park and Han, 2023; Seong and Kim, 2022; Woo, 2021). In competitive educational contexts such as Korea, parental expectations often manifest as achievement pressure, which can foster academic motivation but may also generate stress and anxiety when excessive (Woo, 2021).

Moreover, the ability to establish harmonious school life, maintain stable and satisfactory interpersonal relationships, and experience respect and understanding from others has been shown to play a crucial role in fostering healthy self-esteem (Branden, 2015). Self-regulated learners exhibit heightened awareness of their abilities, intrinsic motivation to learn, and the capacity to monitor and adapt their behavior to environmental demands, thereby promoting smoother adjustment within the school context (Lee and Lee, 2009).

Collectively, these findings indicate that adolescents’ school adjustment is comprehensively shaped by the interplay of parental influences and individual characteristics, making it an essential focal point for ensuring both healthy development and academic success.

Despite substantial evidence linking these variables to school adjustment, few studies have examined the interrelated mechanisms among parental achievement pressure, self-regulated learning, self-esteem, and school adjustment within a single integrative model. To address this gap, the present study investigates the effects of parental achievement pressure and self-regulated learning on adolescents’ school adjustment, with particular attention to the mediating role of self-esteem. By empirically testing this mediating mechanism, the study aims to advance scholarly understanding of the dynamic interaction between parental influences and students’ psychological resources. Furthermore, the findings are expected to yield practical implications by informing the development of psychological and educational intervention strategies that can foster adolescents’ positive adjustment to school life.

The research questions of this study are as follows.

1. What are the relationships among parental achievement pressure, students’ self-regulated learning, self-esteem, and school adjustment?
2. What is the mediating effect of self-esteem on the relationship between parental achievement pressure and students’ self-regulated learning?

### 1.1 The relationship between parental achievement pressure, self-regulated learning, and school adjustment

Parental achievement pressure refers to the academic expectations and evaluative demands that parents place on their children regarding academic success. Within the cultural context of Korean society, this factor is particularly salient, as academic performance has long been regarded as a critical determinant of social mobility and individual status (Kang et al., 2021). Parental achievement pressure can therefore be understood as an evaluative parental attitude that emphasizes academic outcomes as the primary indicator of success. Although moderate levels of parental expectations may function as a motivational factor that encourages students to engage in academic tasks, excessive pressure can generate significant psychological burdens. Previous studies have shown that heightened parental achievement pressure is associated with emotional anxiety, academic stress, and difficulties in overall school life (Cha, 2024; Chen et al., 2009; Woo, 2021). Peng et al. (2025) found that parental achievement pressure—undermines children’s academic self-efficacy, which in turn predicts lower achievement. Similarly, Harma et al. (2025) found that different forms of parental control exert distinct effects on adolescents’ development: while behavioral control—such as parental monitoring and rule-setting—was associated with better self-regulation and adjustment, psychological control characterized by guilt induction, manipulation, and conditional regard showed negative associations with students’ well-being. Empirical evidence supports this concern. For example, Hong and Rho (2022) reported that students’ school adjustment declined significantly as parental achievement pressure increased, suggesting that heightened parental expectations may undermine school adjustment by intensifying anxiety and withdrawal rather than fostering intrinsic academic motivation.

Taken together, these findings indicate that parental achievement pressure functions as a critical contextual factor influencing adolescents’ school adjustment, with excessive pressure likely exerting a negative effect on students’ psychological well-being and adaptation to school life. Accordingly, the present study proposes the following hypothesis:

*Hypothesis 1. Parental achievement pressure has a negative impact on adolescents’ school adjustment.*

Another key factor influencing school adjustment is self-regulated learning. Self-regulated learning refers to learners’ capacity to set academic goals, select and adjust learning strategies, and monitor their own learning processes (Zimmerman, 2000). Self-regulated learning provides an additional lens through which to understand these dynamics. Students who develop strong self-regulated learning skills—including goal setting, strategic planning, and self-monitoring—exhibit greater resilience to the pressures imposed by parental expectations (Xin et al., 2024). This concept extends beyond the mere execution of academic tasks, encompassing the broader ability of learners to actively manage and direct their own learning. Prior research has consistently highlighted its importance. For instance, Noh et al. (2014) found that higher levels of self-regulated learning were associated with greater school adjustment, while Park and Han (2023) demonstrated that self-regulated learning not only enhanced academic achievement but also facilitated broader aspects of school adjustment, including peer relationships, among middle school students. A study that applied self-regulated learning programs to middle school students reported that the school adjustment of the experimental group was significantly higher than that of the control group (Yoon et al., 2011). This aligns with findings in adolescent populations, where perceptions of parental academic involvement and associated stress can exacerbate internalizing problem behaviors, yet psychological resilience and personal value orientations, such as materialism, can mediate these effects (Xue, 2025). Collectively, these studies suggest that self-regulated learning may serve as a protective mechanism that influence the overall effect of parental achievement pressure on school adjustment.

Recent research continues to reinforce the importance of self-regulated learning in contemporary educational contexts. Self-regulated learning promotes students’ academic engagement, motivation, and adaptive learning behaviors, enabling them to effectively cope with academic challenges and adjust to school environments (Šašić et al., 2025). Empirical evidence further indicates that self-regulated learning contributes to improved educational outcomes by fostering sustained learning engagement and strategic learning behaviors (Ouyang, 2025). These findings suggest that students with stronger self-regulated learning capacities are better able to manage academic demands and maintain positive interactions within the school context. In addition, recent work suggests that self-regulated learning processes enable students to effectively manage complex learning tasks, sustain motivation, and adapt to evolving educational environments (Ge et al., 2025).

Taken together, these findings indicate that self-regulated learning plays a critical role in facilitating students’ successful adaptation to school life. Accordingly, the present study proposes the following hypothesis:

*Hypothesis 2. Self-regulated learning has a positive impact on adolescents’ school adjustment.*

Self-esteem can be defined as an individual’s self-evaluation that acknowledges oneself as a positive and valuable being. Students with high self-esteem demonstrate confidence in performing academic tasks, actively engage in learning activities, and experience stable and constructive interactions with both peers and teachers. In contrast, students with low self-esteem often lack confidence in academic contexts, become more vulnerable to academic stress and anxiety, and tend to exhibit withdrawal or marginalization in peer relationships. Such low levels of self-esteem are associated with heightened academic stress, emotional anxiety, and feelings of alienation, which in turn exert a negative influence on school adjustment (Park and Kim, 2015; Jeong and Ahn, 2023b).

Recent research has further highlighted the important role of self-esteem in adolescents’ psychological well-being and school adjustment. Higher levels of self-esteem are positively associated with students’ academic engagement, emotional stability, and social relationships within school contexts (Zhao et al., 2021). Students with stronger self-esteem tend to display greater resilience and adaptive coping strategies when encountering academic challenges, which contributes to better school adjustment. Conversely, diminished self-esteem may weaken students’ motivation and emotional stability, thereby increasing the likelihood of withdrawal and maladaptive responses in school settings. Self-esteem plays an important role in shaping adolescents’ academic engagement and school adjustment. Li et al. (2025) demonstrated that self-esteem mediates the relationship between social interaction styles and school adjustment among secondary school students. Moreover, self-esteem has been identified as a key protective factor that promotes adolescents’ psychological well-being and resilience in school contexts (Yan et al., 2024; Qi et al., 2024). These findings suggest that adolescents with higher self-esteem are better able to regulate their emotions, cope with academic stress, and maintain positive relationships with peers and teachers.

Accordingly, self-esteem functions as a key psychological factor that fosters school adjustment, enabling students to cope effectively with the demands of academic and social life. On the other hand, diminished self-esteem makes school adjustment particularly challenging, given its links to stress, anxiety, and impaired interpersonal relationships. Therefore, self-esteem can be regarded as a crucial variable in predicting adolescents’ school adjustment. Based on this rationale, the following hypothesis is proposed:

*Hypothesis 3. Self-esteem has a positive impact on adolescents’ school adjustment.*

Parental achievement pressure is closely associated with adolescents’ self-esteem. Excessive parental emphasis on academic success can negatively influence self-esteem, as children may come to evaluate their self-worth primarily in terms of grades and test performance (Hwang and Jung, 2012). In this context, high self-esteem may buffer the negative effects of parental achievement pressure, whereas low self-esteem can function as a risk factor that increases susceptibility to academic stress. Empirical evidence supports this claim: Son and Jung (2021) reported that higher levels of parental achievement pressure were significantly associated with lower levels of adolescents’ self-esteem. Empirical studies have shown that strong parental expectations and performance-oriented parenting styles are associated with increased academic stress and lower levels of self-esteem among adolescents (Yan et al., 2024). And excessive monitoring, and achievement-focused expectations can negatively influence adolescents’ self-esteem by fostering feelings of inadequacy and fear of failure (Liu et al., 2025). Similarly, recent findings suggest that adolescents exposed to high levels of parental academic pressure are more likely to experience diminished self-worth and psychological distress, especially when academic performance becomes the primary criterion for parental approval (Qi et al., 2024).

These findings suggest that parental achievement pressure may erode adolescents’ self-esteem by reinforcing performance-based self-evaluation and increasing sensitivity to academic failure. Conversely, adolescents with stronger self-esteem may be better able to cope with parental expectations and academic challenges. Taken together, the existing literature indicates that excessive parental achievement pressure may undermine adolescents’ self-esteem. Accordingly, the present study proposes the following hypothesis:

*Hypothesis 4. Parental achievement pressure has a negative impact on adolescents’ self-esteem.*

Self-regulated learning is closely related to the development of self-esteem during adolescence. Self-regulated learning refers to learners’ ability to set learning goals, select and implement appropriate strategies, and monitor and regulate their own learning processes (Zimmerman, 2000). During adolescence, academic achievement becomes an increasingly important domain in which students evaluate their competence and personal value. Accordingly, the development of effective self-regulatory learning strategies may contribute positively to adolescents’ self-esteem by enabling them to successfully manage academic tasks and achieve learning goals. Students who possess stronger self-regulated learning skills are more likely to experience mastery and competence in academic settings, which can foster greater confidence and self-efficacy, ultimately leading to higher levels of self-esteem (Kim, 2017; Lim, 2013).

Recent studies have continued to emphasize the importance of self-regulated learning as a key psychological resource in adolescents’ academic and socio-emotional development. For example, research has shown that students with stronger self-regulatory capacities tend to experience higher levels of mastery, competence, and academic confidence, which are closely associated with positive self-perceptions and self-worth (Panadero, 2017). In other words, as the academic domain increasingly constitutes a central component of self-esteem during the school years, self-regulated learning within academic contexts can be regarded as an important factor in the development of self-esteem. Supporting this view, Woo (2014) confirmed that the self-regulated learning skill was significant factors in explaining children’s self-esteem. And adolescents with higher levels of self-regulated learning reported stronger feelings of mastery and self-efficacy, which foster positive emotional experiences and reinforce confidence in their academic abilities (Sun and Zhang, 2026). Furthermore, studies have shown that self-regulation is closely linked to psychological well-being and adaptive functioning among students. Self-regulated learners tend to exhibit stronger self-efficacy and emotional regulation, which contribute to improved psychological well-being and positive learning experiences (Fan and Cui, 2024).

Building on the theoretical and empirical evidence discussed above, self-regulated learning appears to play an important role in shaping adolescents’ self-evaluations within academic contexts. By enabling students to set goals, monitor their learning processes, regulate effort, and apply effective learning strategies, self-regulated learning facilitates successful academic experiences and strengthens students’ perceptions of competence. These mastery experiences and enhanced feelings of self-efficacy can, in turn, promote more positive self-perceptions and contribute to the development of higher self-esteem during adolescence. Therefore, self-regulated learning can be understood as a key psychological mechanism that supports both academic functioning and the formation of positive self-evaluations among adolescents.

Based on this theoretical reasoning and empirical evidence, the present study proposes the following hypothesis:

*Hypothesis 5. Self-regulated learning has a positive impact on adolescents’ self-esteem.*

### 1.2 The mediating role of self-esteem

A growing body of research suggests that adolescents’ school adjustment is influenced not only by external environmental factors but also by internal psychological resources that shape how students respond to academic demands and expectations. In particular, parental achievement pressure has been widely discussed as an important contextual factor affecting adolescents’ adjustment to school life. Previous studies indicate that parental achievement pressure does not always exert a direct effect on school adjustment; rather, its influence often operates indirectly through psychological mechanisms and individual characteristics (Choi and Han, 2021; Hong and Rho, 2022; Hae, 2022; Jung and Kim, 2019; Kim, 2022; Yeon and Choi, 2022). Excessive parental expectations and achievement-oriented pressure may create psychological burdens for adolescents, leading to increased stress, emotional tension, and negative self-perceptions, which in turn hinder their ability to adapt successfully to school environments.

In other words, parental achievement pressure may induce negative psychological states such as stress in children, which can serve as risk factors for successful school adjustment. Within this relationship, children’s internal factors—most notably self-esteem—can operate as mediating variables. Parental achievement pressure has been shown to negatively affect children’s school adjustment, with child autonomy serving as a significant mediating factor (Hong and Rho, 2022). Empirical evidence has also shown that self-esteem significantly predicts various indicators of school adjustment, including academic participation, interpersonal relationships, and satisfaction with school life (Lim, 2013). Recent studies further suggest that self-esteem frequently functions as a mediating variable linking family environment factors and adolescents’ adjustment outcomes.

Recent studies have continued to emphasize the importance of self-esteem as a psychological mechanism through which contextual and interpersonal factors influence adolescents’ developmental outcomes. For instance, self-esteem significantly mediates the relationship between interpersonal characteristics and students’ school adjustment, suggesting that adolescents with higher self-esteem are more likely to cope effectively with academic and social challenges in school environments (Tang et al., 2025). Furthermore, other recent studies have indicated that self-esteem is closely associated with adolescents’ mental health, resilience, and coping strategies, reinforcing its role as an important psychological resource that supports positive developmental trajectories (Chen et al., 2025; Liao et al., 2024). The relationship between parental achievement pressure and school adjustment appears to be mediated by self-esteem, which functions as an internal resource for coping with academic stress. Jung and Ahn (2023) demonstrated that family processes, including supportive parenting and caring, contribute to higher self-esteem, which in turn facilitates better school adjustment among middle school students. In contrast, academic stress, intensified by performance-oriented parenting, has been negatively linked to school adjustment, although participation in school-based activities, such as sports clubs, may buffer these effects (Jung and Kim, 2019). In elementary school populations, structural analyses indicate that parenting stress and achievement pressure significantly predict school adjustment outcomes, reinforcing the critical mediating role of children’s self-esteem in mitigating negative academic and social effects (Min, 2024).

At the same time, self-regulated learning has been widely recognized as an important individual capability that contributes to both academic success and psychological development. Students who effectively regulate their learning processes tend to set clear learning goals, monitor their progress, and apply adaptive learning strategies. These experiences foster a stronger sense of competence and self-efficacy, which can enhance positive self-evaluations and strengthen students’ self-esteem (Panadero, 2017). In addition, self-esteem and self-regulated learning jointly contribute to students’ academic engagement and positive adjustment outcomes (Zhao et al., 2021). These findings suggest that self-regulated learning may influence school adjustment not only directly but also indirectly by strengthening internal psychological resources such as self-esteem.

Taken together, self-esteem can be conceptualized as an important mediating mechanism in the relationships among parental achievement pressure, self-regulated learning, and adolescents’ school adjustment. Accordingly, this study proposes the following hypotheses:

*Hypothesis 6. Self-esteem mediates the relationship between achievement pressure and school adjustment.*

*Hypothesis 7. Self-esteem mediates the relationship between self-regulated learning and school adjustment.*

## 2 Research methods

### 2.1 Participants and procedures

Data for this study were drawn from the 14th wave (2021) of the *Panel Study on Korean Children (PSKC)*, a nationally representative longitudinal survey. Of the 2,150 13-year-old students surveyed, a total of 1,328 were included in the present analysis after excluding cases with incomplete data. The sample comprised approximately equal proportions of boys and girls. All participants and their parents provided informed consent prior to data collection. This study used secondary data from the Panel Study on Korean Children (PSKC), which was conducted under the approval of the National Research Council for Economics, Humanities and Social Sciences (Korea). All procedures complied with relevant ethical guidelines and regulations.

### 2.2 Measures

#### 2.2.1 Parental achievement pressure scale

Parental achievement pressure was assessed using a questionnaire comprising 6 items on behavioral constraints (e.g., “*My parents do not like me to go out with friends without studying*”), 3 items on grade management (e.g., “*My parents are most interested in my academic performance*”), and 6 items on academic guidance (e.g., “*My parents tell me that studying is the most important*”). Responses were recorded on a 5-point Likert scale (1 = strongly disagree, 5 = strongly agree), with lower scores indicating lower parental achievement pressure and higher scores indicating higher parental achievement pressure. The internal consistency reliability in this study was 0.94.

#### 2.2.2 Self-regulated learning scale

Self-regulated learning was assessed using a questionnaire comprising 2 items on overcoming difficulties (e.g., “*I spend more time and effort when studying difficult subjects*”) and 3 items on exploring study methods (e.g., “*I monitor my understanding and effort while studying*”). Responses were recorded on a 4-point Likert scale (1 = strongly disagree, 4 = strongly agree), with lower scores indicating lower self-regulated learning and higher scores indicating higher self-regulated learning. The internal consistency reliability in this study was 0.81, meeting the commonly accepted threshold for adequate reliability (*α* ≥ 0.70) (Hair et al., 2019). Although the scale consists of only five items, recent research in educational psychology suggests that brief scales reflecting core strategies can still achieve sufficient reliability and construct validity. In particular, in the assessment of self-regulated learning, items capturing key behavioral strategies—such as effort regulation and metacognitive monitoring—are considered central indicators of the construct (Panadero, 2017). Furthermore, recent validation research has demonstrated that short versions of self-regulated learning instruments can also be used as valid and efficient measures in educational research contexts (Wang et al., 2024). Therefore, the items used in this study can be considered to appropriately capture the construct, as they reflect core strategies of self-regulated learning.

#### 2.2.3 Self-esteem scale

Self-esteem was assessed with a total of five items, which were divided into two subdimensions: self-satisfaction (3 items) and self-evaluation (2 items). Self-satisfaction reflects the extent to which students feel content and positive about themselves (e.g., *“I am satisfied with myself”*). Self-evaluation captures students’ subjective appraisal of their own worth and abilities (e.g., *“I feel that I have a number of good qualities”*). Responses were recorded on a 4-point Likert scale (1 = strongly disagree, 4 = strongly agree), with lower scores indicating lower self-esteem and higher scores indicating higher self-esteem. The internal consistency reliability in this study was 0.86.

#### 2.2.4 School adjustment scale

School adjustment was measured using 38 items, comprising four subdimensions: school studies (8 items), school friends (10 items), school teachers (10 items), and school life (10 items). The school studies dimension reflects the student’s ability to engage with and manage academic tasks (e.g., *“I try to complete my school assignments on time”*). School friends assess the quality of interactions and friendships with classmates (e.g., *“I get along well with my classmates”*). School teachers capture the degree of trust and rapport students have with teachers (e.g., *“I feel comfortable talking to my teachers when I need help”*). Finally, school life reflects the overall sense of belonging and satisfaction with daily school experiences (e.g., *“I enjoy participating in school activities”*). Responses were recorded on a 5-point Likert scale (1 = strongly disagree, 5 = strongly agree), with lower scores indicating lower school adjustment and higher scores indicating higher school adjustment. The internal consistency reliability in this study was 0.94.

### 2.3 Data analysis

#### 2.3.1 Data analysis methodology

In this study, a structural equation model (SEM) was employed to examine the relationships among parental achievement pressure, self-regulated learning, self-esteem, and school adjustment. Data analysis was conducted using SPSS 27.0 and R 4.4.1. The specific analytical procedures were as follows. First, descriptive statistics were calculated for the measurement instruments, including parental achievement pressure, self-regulated learning, self-esteem, and school adjustment. Second, Pearson correlation coefficients were computed to examine the relationships among these variables, and their statistical significance was evaluated. Third, the goodness-of-fit of the research model was assessed using *χ*^2^, Comparative Fit Index (CFI), Tucker–Lewis Index (TLI), Root Mean Square Error of Approximation (RMSEA), and Standardized Root Mean Square Residual (SRMR). Fourth, confirmatory factor analysis (CFA) and path analysis were performed to investigate the effects of achievement pressure and self-regulated learning on school adjustment. Finally, bootstrapping was conducted to examine the effects of parental achievement pressure and self-regulated learning on school adjustment, as well as the mediating role of self-esteem. The mediating role of self-esteem was examined using the bootstrap method with 5,000 resamples, with indirect effects considered significant if the 95% confidence interval did not include zero.

Gender and school type were collected as part of the participants’ demographic information and were included as control variables in the structural equation model. These variables were incorporated as covariates to account for potential demographic influences on the relationships among the key constructs. Controlling for demographic factors such as gender and school type is recommended in structural equation modeling when these characteristics may be associated with students’ learning experiences or adjustment outcomes (Hair et al., 2019; Kline, 2016). Accordingly, gender and school type were entered into the SEM analysis to statistically control for their potential effects while examining the structural relationships among the main constructs—parental pressure, self-regulated learning, self-esteem, and school adjustment. Gender and school type were recorded as part of the participants’ demographic characteristics.

**Figure 1:**
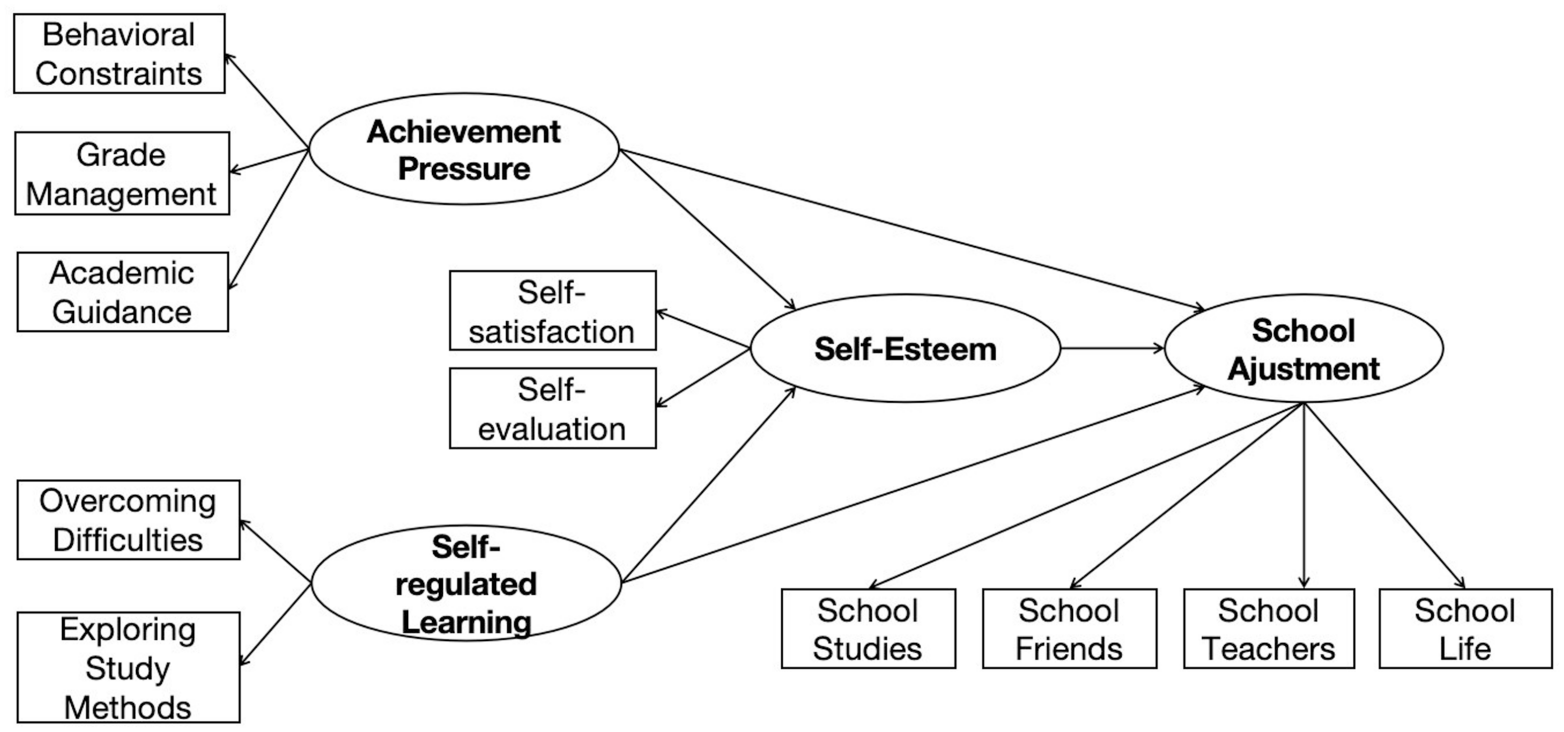
Research model.

The research model for this study is presented in Figure 1.

## 3 Results

### 3.1 Descriptive statistics and correlation analysis

The results of the descriptive statistical analysis for the main study variables—parental achievement pressure, self-regulated learning, self-esteem, and school adjustment—are presented in Table 1. The skewness values ranged from −0.584 to 0.546, and the kurtosis ranged from −0.410 to 1.219, all within acceptable thresholds (|skewness| < 2, |kurtosis| < 7), indicating that the assumption of normality was satisfied (Kline, 2005).

**Table 1:** Descriptive statistics of measurement variables (*N* = 1,328).

Variable		Mean (SD)	Min	Max	Skewness	Kurtosis
Gender		1.48(0.013)	1	2	0.67	0.134
School Type		1.07(0.007)	1	2	0.067	0.134
Achievement pressure	Behavioral constraints	2.27(0.83)	1	5	0.472	−0.130
	Grade management	2.15(0.95)	1	5	0.546	−0.355
	Academic guidance	2.53(0.81)	1	5	0.233	−0.410
Self-regulated learning	Overcoming Difficulties	2.76(0.58)	1	4	−0.552	0.808
	Exploring study methods	2.51(0.61)	1	4	−0.134	0.267
Self-esteem	Self-satisfaction	3.17(0.57)	1	4	−0.379	0.336
	Self-evaluation	3.22(0.55)	1	4	−0.429	0.560
School adjustment	School studies	3.69(0.64)	1	5	−0.222	0.478
	School friends	4.10(0.61)	1	5	−0.584	0.356
	School teachers	3.62(0.73)	1	5	−0.354	0.434
	School life	4.01(0.59)	1	5	−0.556	1.219

The results of the correlation analysis among the main study variables are presented in Table 2. First, the correlation between achievement pressure and self-regulated learning was *r* = 0.130, indicating a statistically significant but relatively weak positive relationship. This suggests that students who experience high achievement pressure tend to exhibit slightly higher self-regulated learning behaviors. The relationship between achievement pressure and self-esteem was *r* = −0.142, demonstrating a statistically significant negative correlation. This finding implies that higher levels of achievement pressure are associated with lower levels of self-esteem among adolescents. Similarly, the correlation between achievement pressure and school adjustment was *r* = −0.132, which was also statistically significant and negative, suggesting that students who experience higher achievement pressure tend to have slightly lower levels of adjustment to the school environment. Regarding self-regulated learning, the correlation with self-esteem was *r* = 0.264, indicating a statistically significant positive relationship. This suggests that students who engage more actively in self-regulatory learning tend to exhibit higher self-esteem. Finally, the correlation between self-esteem and school adjustment was *r* = 0.582, representing a strong positive association. This finding highlights that adolescents with higher self-esteem are more likely to adapt well to their school environment. All correlations were below 0.90, indicating no multicollinearity concerns and supporting the inclusion of these variables in subsequent SEM analyses.

**Table 2:** Correlations among main study variables (*N* = 1,328).

Variables	Gender	School type	Achievement pressure	Self-regulated learning	Self-esteem	School adjustment
Gender	1					
School type	−0.01	1				
Achievement pressure	0.024	0.04	1			
Self-regulated learning	0.007	−0.005	0.130***	1		
Self-esteem	0.028	−0.01	−0.142***	0.264***	1	
School adjustment	0.026	−0.001	−0.132***	0.550***	0.582***	1

^*^p < 0.05, ^**^p < 0.01, ^***^p < 0.001.

As shown in Table 1, Gender was coded as 1 = male and 2 = female (*M* = 1.48, SD = 0.013), indicating a relatively balanced gender distribution. School type was coded as 1 = public school and 2 = private school (*M* = 1.07, SD = 0.007), suggesting that the majority of students attended public schools.

As shown in Table 2, gender showed very small and non-significant correlations with the key constructs, including achievement pressure (*r* = 0.024), self-regulated learning (*r* = 0.007), self-esteem (*r* = 0.028), and school adjustment (*r* = 0.026). These results suggest that gender differences in the main psychological variables examined in this study were minimal within the present sample, indicating that the observed effects are less likely to be confounded by these demographic factors. Nevertheless, following the reviewer’s suggestion, gender was included as a control variable in the structural equation model to account for potential demographic influences. By statistically controlling for gender, the analysis ensured that the estimated relationships among achievement pressure, self-regulated learning, self-esteem, and school adjustment were not confounded by gender differences.

### 3.2 Structural equation model analysis

#### 3.2.1 Model fit

The hypothesized structural equation model demonstrated an overall good fit to the data. The overall goodness-of-fit of the model is presented in Table 3.

**Table 3:** Research model fit index.

Division	$x^{2}$ (*df*)	CFI	TLI	RMSEA (90% CI)	SRMR
Fit indices	253.427(38)^***^	0.970	0.957	0.065 (0.058 ~ 0.073)	0.039

^*^p < 0.05, ^**^p < 0.01, ^***^p < 0.001.

The Chi-square (*χ*^2^) test was used as the basic indicator of model-data correspondence. Although *χ*^2^ is a widely employed measure, it is known to be highly sensitive to sample size, often leading to the rejection of models even when the fit is acceptable in practical terms (Kline, 2005). For this reason, it is recommended to consider additional fit indices that are less affected by sample size and the number of estimated parameters. In this study, model adequacy was therefore further evaluated using the Comparative Fit Index (CFI), the Tucker–Lewis Index (TLI), the Root Mean Square Error of Approximation (RMSEA), and the Standardized Root Mean Square Residual (SRMR).

The Comparative Fit Index (CFI) and Tucker–Lewis Index (TLI) are incremental fit indices in which higher values indicate better model fit, with values above 0.90 generally considered acceptable and values above 0.95 indicating excellent fit (Kline, 2005). In this study, the TLI was 0.970 and the CFI was 0.957, both suggesting a very good model fit. RMSEA is a parsimony adjustment index that evaluates a simpler model as a good model among models with similar explanatory power in relation to the same data. Generally, a value of 0.06 or less is considered a good level of fit, and a value of 0.08 or less is considered a good level of fit (Browne and Cudeck, 1993). The RMSEA for this model was 0.065, with a 90% confidence interval of 0.058–0.073, demonstrating an acceptable level of fit. Furthermore, the SRMR was 0.039, which falls below the recommended threshold of 0.06. Taken together, these results provide strong support for the adequacy of the hypothesized model.

#### 3.2.2 Path analysis of the research model

**Figure 2:**
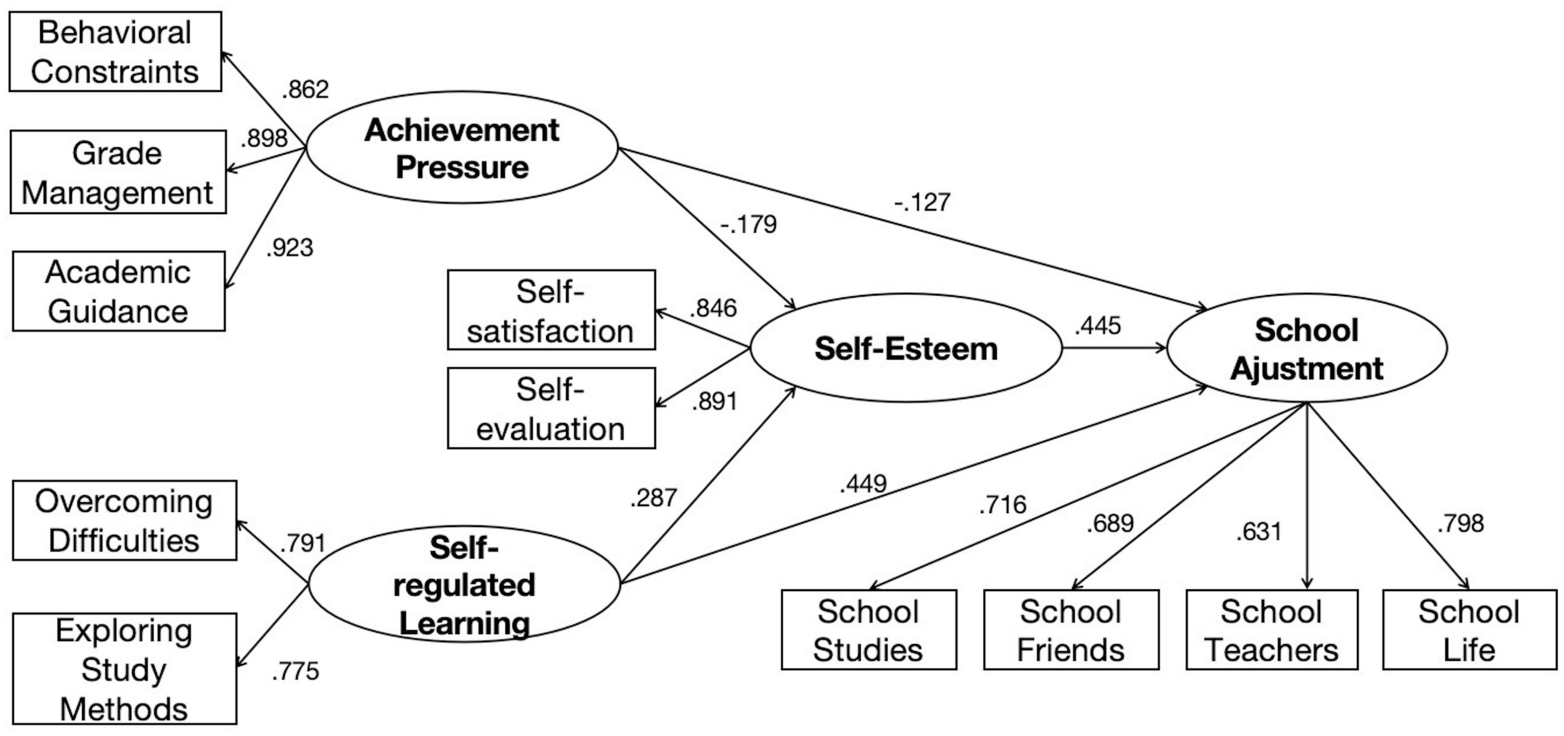
SEM model.

Table 4 and Figure 2 presents the parameter estimates for the hypothesized structural model. Achievement pressure negatively predicted school adjustment (*β* = −0.127, *p* < 0.001), indicating that higher levels of achievement pressure are associated with lower levels of school adjustment. And achievement pressure demonstrated a negative effect on self-esteem (*β* = −0.179, *p* < 0.001), indicating that higher achievement pressure corresponds to lower self-esteem. In contrast, self-regulated learning had significant positive effects on school adjustment (*β* = 0.449, *p* < 0.001) and self-esteem (*β* = 0.287, *p* < 0.001). Finally, self-esteem positively predicted school adjustment (*β* = 0.445, *p* < 0.001). All coefficients were statistically significant at the 0.001 level. Taken together, these findings indicate that all hypothesized paths were statistically significant, supporting the proposed model. These findings indicate that while parental achievement pressure undermines adolescents’ self-esteem and school adjustment, self-regulated learning fosters resilience by strengthening self-esteem, which in turn facilitates smoother adaptation to school contexts.

**Table 4:** Standardized path coefficients for the structural model.

Regression equation	Path	Unstandardized coefficient	Standardized coefficient (*β*)
Structure coefficient	Achievement pressure → School adjustment	−0.081^***^	−0.127
	Self-regulated learning → School adjustment	0.447^***^	0.449
	Self-esteem → School adjustment	0.423^***^	0.445
	Achievement pressure → Self-esteem	−0.121^***^	−0.179
	Self-regulated learning → Self-esteem	0.301^***^	0.287
Factor loading	Achievement pressure → Behavioral constraints	1	0.862
	Achievement pressure → Overcoming difficulties	1.192^***^	0.898
	Achievement pressure → Exploring study methods	1.146^***^	0.923
	Self-regulated learning → Self-satisfaction	1	0.791
	Self-regulated learning → Self-assessment	1.027^***^	0.775
	Self-esteem → Self-satisfaction	1	0.846
	Self-esteem → Self-assessment	1.012^***^	0.891
	School adjustment → School study	1	0.716
	School adjustment → School friends	0.920^***^	0.689
	School adjustment → School teachers	1.012^***^	0.631
	School adjustment → School life	1.034^***^	0.798

^*^p < 0.05, ^**^p < 0.01, ^***^p < 0.001.

### 3.3 Analysis of the mediating effect of self-esteem

This study employed bootstrapping to examine the statistical significance of the mediating role of self-esteem in the relationship between achievement pressure, self-regulatory learning, and school adjustment, with a 95% confidence interval. The bootstrapped estimates of the mediation effect are presented in Table 5, while the direct, indirect, and total effects between the variables, along with their significance levels, are summarized in Table 6.

**Table 5:** Mediation effect verification.

Path	95% CI (lower – upper)	Mediation
Achievement pressure → Self-esteem → School adjustment	−0.077–0.026	Yes
Self-regulated learning → Self-esteem → School adjustment	0.111 0.228	Yes

^*^p < 0.05, ^**^p < 0.01, ^***^p < 0.001.

**Table 6:** Direct/indirect effects and total effect.

Path	Direct effect	Indirect effect	Total effect
Achievement pressure → School adjustment	−0.127^***^	−0.052^***^	−0.179^***^
Self-regulated learning → School adjustment	0.449^***^	0.167^***^	0.616^***^
Self-esteem → School adjustment	0.445^***^	–	0.445^***^
Achievement pressure → Self-esteem	−0.179^***^	–	−0.179^***^
Self-regulated learning → Self-esteem	0.287^***^	–	0.287^***^

^*^p < 0.05, ^**^p < 0.01, ^***^p < 0.001.

Parental achievement pressure had a significant total effect on school adjustment (*β* = −0.179, *p* < 0.001). The direct path remained negative and substantial (*β* = −0.127, *p* < 0.001), accounting for about 71% of the total effect, while the indirect effect through self-esteem was also significant (*β* = −0.052, 95% CI [−0.077, −0.026], *p* < 0.001), representing 29% of the total effect. These results suggest that part of the adverse impact of parental achievement pressure on school adjustment operates by lowering adolescents’ self-esteem.

Self-regulated learning, by contrast, exerted a strong positive total effect on school adjustment (*β* = 0.616, *p* < 0.001). The direct effect (*β* = 0.449, *p* < 0.001) explained 73% of the total effect, while the indirect effect through self-esteem (*β* = 0.167, 95% CI [0.111, 0.228], *p* < 0.001) accounted for the remaining 27%. This indicates that adolescents who engage in higher levels of self-regulated learning not only adapt better directly, but also benefit indirectly through enhanced self-esteem, which further facilitates adjustment.

Some effects, such as the influence of parental pressure on school adjustment (*β* = −0.127), were statistically significant but relatively modest in magnitude. From this perspective, the effect of parental pressure on school adjustment can be interpreted as statistically meaningful but practically limited. Previous studies have also suggested that excessive parental pressure may negatively influence students’ psychological well-being and adjustment, although its direct impact on school adjustment tends to be relatively small compared with internal learning factors (Pomerantz and Wang, 2009). For example, research has shown that controlling or pressuring parental behaviors are often associated with lower levels of autonomy and psychological adjustment among students, but their direct predictive power for school adjustment outcomes tends to be modest (Eccles, 2007; Hill and Tyson, 2009).

In contrast, the effect of self-directed learning on school adjustment (*β* = 0.449) appears substantially stronger, indicating a moderate to relatively large effect. This finding is consistent with a substantial body of literature emphasizing the central role of self-directed or self-regulated learning in students’ academic engagement, persistence, and adaptation to school environments (Zimmerman, 2002; Pintrich, 2004). Self-directed learning enables students to set learning goals, monitor their progress, regulate learning strategies, and maintain motivation when encountering academic challenges. These processes are closely related to students’ ability to adapt to school life and effectively manage academic demands (Zimmerman and Schunk, 2011; Broadbent and Poon, 2015). Therefore, compared with external contextual factors such as parental pressure, self-directed learning may exert a stronger influence on school adjustment because it directly supports students’ active engagement with learning tasks and their capacity to cope with academic difficulties.

In addition, self-esteem itself significantly predicted school adjustment (β = 0.445, *p* < 0.001), underscoring its role as a critical psychological resource. Taken together, these findings confirm that self-esteem partially mediates the effects of both parental achievement pressure and self-regulated learning on adolescents’ school adjustment, functioning as a key pathway through which environmental risk and protective factors shape adjustment outcomes.

## 4 Discussion

### 4.1 Relationship between parental achievement pressure, self-regulated learning, self-esteem, and school adjustment

The purpose of this study was to examine the structural relationships among parental achievement pressure, self-regulated learning, self-esteem, and school adjustment, and to analyze whether children’s self-esteem functions as a mediator in the process through which parental achievement pressure and self-regulated learning influence school adjustment. The key findings of this study can be summarized as follows.

First, parental achievement pressure exerted a significant influence on adolescents’ school adjustment. Consistent with prior studies (Cha, 2024; Hong and Rho, 2022; Ha, 2022; Kim, 2022), heightened parental expectations tended to hinder students’ adaptation by increasing stress and reducing autonomy. For children to adjust successfully to school, it is important that parents refrain from imposing excessive academic demands and instead place greater emphasis on fostering emotional stability and personal autonomy. Since children develop at different rates depending on individual characteristics, excessive parental pressure can hinder healthy growth and adjustment. Accordingly, it is necessary to develop tailored counseling interventions and parent–child communication programs for families experiencing high levels of achievement pressure. From an international perspective, the influence of parental achievement pressure should also be interpreted within broader cultural and educational contexts. In many East Asian societies, including Korea, China, and Japan, academic achievement is often strongly emphasized because educational success is perceived as a critical pathway to social mobility and future career opportunities. Consequently, parents in these contexts tend to place relatively high expectations on their children’s academic performance (Chen et al., 2014). Although such expectations may sometimes serve as a motivational factor for students, excessive parental pressure can increase psychological stress and negatively affect students’ emotional well-being and school adjustment. In contrast, in many Western educational contexts, parenting practices tend to place greater emphasis on children’s autonomy, intrinsic motivation, and individual interests. Parental involvement in education is therefore more likely to focus on providing emotional support and encouraging self-directed learning rather than exerting direct academic pressure (Hill and Tyson, 2009). As a result, the relationship between parental achievement pressure and school adjustment may manifest differently across cultural contexts. These differences suggest that parenting values and educational systems play an important role in shaping how parental expectations influence adolescents’ psychological development and school experiences.

Second, it was found that self-regulated learning among middle school students has a significant positive effect on school adjustment. This finding is consistent with numerous previous studies, which have demonstrated that higher levels of self-regulated learning are associated with greater school adjustment (Jeong and Choi, 2018; Lim, 2013; Noh et al., 2014; Park and Han, 2023; Yoon et al., 2011). This can be interpreted to mean that when students actively monitor and control their own learning processes, they not only enhance academic performance but also improve emotional regulation and interpersonal relationships, thereby facilitating overall school adjustment. In other words, the results suggest that strengthening self-regulated learning skills is essential for middle school students to successfully adjust to school life. Numerous international studies have shown that students with higher levels of self-regulated learning tend to demonstrate stronger academic achievement, greater motivation, and better adaptation to school environments (Zimmerman and Schunk, 2011; Dent and Koenka, 2016). In addition, cross-national educational assessments have highlighted the importance of self-regulated learning as a key competency for successful learning in modern educational systems (OECD, 2019). These findings suggest that self-regulated learning functions not only as a cognitive learning strategy but also as an important psychological resource that helps students manage academic demands and adapt effectively to school life across diverse educational contexts.

Third, the analysis of the relationship between parental achievement pressure and middle school students’ self-esteem revealed that higher levels of parental achievement pressure were associated with lower levels of self-esteem among students. In other words, when parents place disproportionate importance on academic performance, students may experience heightened psychological burdens and academic stress, which in turn undermine their self-worth. Previous research suggests that parenting practices characterized by excessive control, conditional approval, or performance-oriented expectations may negatively affect children’s psychological well-being and self-worth (Assor et al., 2004; Soenens and Vansteenkiste, 2010). Therefore, it is important to enhance students’ self-esteem by recognizing and praising their efforts in the learning process, respecting their intrinsic needs, and supporting their confidence in academic pursuits, rather than focusing narrowly on achievement outcomes. In many East Asian societies, where academic achievement is strongly emphasized as a pathway to social mobility, parental expectations are often higher than in many Western contexts. Although such expectations can sometimes promote academic motivation, excessive achievement pressure may still increase academic stress and negatively affect adolescents’ emotional well-being and self-perception (Quach et al., 2015). Therefore, international research increasingly emphasizes the importance of autonomy-supportive parenting practices that acknowledge students’ efforts, provide emotional support, and encourage intrinsic motivation rather than focusing solely on academic performance (Soenens and Vansteenkiste, 2010). Such approaches can help students maintain positive self-esteem while coping with academic demands, thereby supporting both their psychological well-being and their long-term educational development.

Fourth, self-regulated learning was found to have a significant positive effect on self-esteem. In other words, the more actively students engage in self-regulated learning, the higher their self-esteem tends to be. This finding is consistent with prior studies that have demonstrated a positive relationship between self-regulated learning and self-esteem (Kim, 2017; Lim, 2013; Woo, 2014). These results indicate that when students adjust their behaviors and attitudes during the learning process and manage their academic performance effectively, they develop greater confidence in their abilities, which subsequently enhances their self-esteem. However, the applicability of these findings should be considered within broader Asian and international educational contexts. In many Asian education systems, including Korea, China, and Japan, learning environments are often characterized by high academic competition and strong societal expectations regarding academic achievement. In such contexts, self-regulated learning may function not only as a learning strategy but also as an important psychological resource that helps students cope with academic pressure and maintain positive self-perceptions. Previous studies have suggested that students who effectively regulate their learning processes are better able to manage academic demands and sustain motivation, which in turn supports the development of positive self-evaluations (Zimmerman, 2002; Dent and Koenka, 2016).

### 4.2 The mediating role of self-esteem

Parental achievement pressure has been shown to have a significant effect on students’ school adjustment, with higher parental expectations for academic performance exerting a negative influence on children’s adjustment to school life. At the same time, self-regulated learning among middle school students was found to have a significant positive effect on school adjustment. Excessive parental emphasis on academic achievement was associated with increased psychological burden and academic stress for students, which in turn lowered their self-esteem. By contrast, self-regulated learning demonstrated a significant positive effect on self-esteem, and self-esteem was confirmed as a statistically significant mediator in the relationship between parental achievement pressure, self-regulated learning, and school adjustment. In other words, when parents emphasize learning outcomes excessively, middle school students may experience academic stress and emotional strain, leading to reduced self-esteem and, consequently, poorer school adjustment. Conversely, students with higher levels of self-regulated learning tend to report stronger self-esteem, as they set their own learning goals, overcome challenges, and employ strategies to relieve stress. This enhanced self-esteem contributes not only to academic achievement but also to smoother adaptation in other areas of school life, including peer and teacher relationships.

Therefore, it is necessary to implement practical intervention strategies that can support students’ self-esteem and self-regulated learning. For example, schools could provide parent education programs that promote positive parenting attitudes, such as workshops on constructive feedback, autonomy-supportive communication, and realistic expectations for children’s academic achievement. In addition, schools and teachers could implement mentoring or counseling programs aimed at strengthening students’ self-esteem. Within the classroom, teachers may also support students’ self-regulated learning by encouraging goal-setting activities, reflective learning journals, and opportunities for students to monitor and evaluate their own learning progress.

### 4.3 Research significance, limitations, and future directions

This study contributes to the literature by clarifying the mechanisms linking parental achievement pressure, self-regulated learning, and school adjustment, highlighting the mediating function of self-esteem. Nevertheless, several limitations warrant consideration.

First, this study utilized only the first-year dataset from the 2021 Korean Children’s Panel. While the cross-sectional analysis yielded meaningful results, it inevitably restricted the ability to capture developmental changes over time. Therefore, the findings should be interpreted as indicating associations rather than cause-and-effect relationships. Future research is encouraged to employ longitudinal designs to examine how parental achievement pressure, self-regulated learning, self-esteem, and school adjustment interact and develop over time, thereby providing a clearer understanding of the potential developmental processes underlying these relationships.

Second, all variables were assessed using self-report questionnaires. Although self-reported data are widely used in social science research, they are often limited by potential biases such as social desirability, memory recall errors, or respondents’ subjective interpretation of items. In particular, the measurement precision of the self-report scales employed in this study may not have been sufficient to capture the full complexity of constructs such as achievement pressure, self-regulated learning, and self-esteem. To address these limitations, future research should make efforts to enhance the validity and reliability of measurements by developing more sophisticated instruments tailored to the specific cultural and developmental contexts of middle school students. Furthermore, future research should consider incorporating multiple sources of data, such as parent or teacher reports as well as behavioral observations, rather than relying solely on self-reported measures. The use of multiple informants and observational approaches may help reduce common method bias and improve the reliability and validity of the conclusions.

Finally, it is essential to enhance the precision of the analysis regarding the relationships among the variables by incorporating appropriate control variables, such as gender and school type, which are theoretically and empirically recognized as potential factors influencing school adjustment, the dependent variable. By controlling for these variables, the study can more rigorously isolate the unique effects of the independent variables on school adjustment, thereby reducing confounding influences and increasing the internal validity of the findings.

## Data Availability

The original contributions presented in the study are included in the article/supplementary material, further inquiries can be directed to the corresponding author.
